# Computational Modeling of the Hematopoietic Erythroid-Myeloid Switch Reveals Insights into Cooperativity, Priming, and Irreversibility

**DOI:** 10.1371/journal.pcbi.1000268

**Published:** 2009-01-23

**Authors:** Vijay Chickarmane, Tariq Enver, Carsten Peterson

**Affiliations:** 1 Division of Biology, California Institute of Technology, Pasadena, California, United States of America; 2 MRC Molecular Biology Unit, Weatherall Institute of Molecular Medicine, University of Oxford, John Radcliffe Hospital, Headington, Oxford, United Kingdom; 3 Lund Strategic Research Center for Stem Cell Biology and Cell Therapy, Lund University, Lund, Sweden; 4 Computational Biology and Biological Physics, Department of Theoretical Physics, Lund University, Lund, Sweden; Emory University, United States of America

## Abstract

Hematopoietic stem cell lineage choices are decided by genetic networks that are turned ON/OFF in a switch-like manner. However, prior to lineage commitment, genes are primed at low expression levels. Understanding the underlying molecular circuitry in terms of how it governs both a primed state and, at the other extreme, a committed state is of relevance not only to hematopoiesis but also to developmental systems in general. We develop a computational model for the hematopoietic erythroid-myeloid lineage decision, which is determined by a genetic switch involving the genes PU.1 and GATA-1. Dynamical models based upon known interactions between these master genes, such as mutual antagonism and autoregulation, fail to make the system bistable, a desired feature for robust lineage determination. We therefore suggest a new mechanism involving a cofactor that is regulated as well as recruited by one of the master genes to bind to the antagonistic partner that is necessary for bistability and hence switch-like behavior. An interesting fallout from this architecture is that suppression of the cofactor through external means can lead to a loss of cooperativity, and hence to a primed state for PU.1 and GATA-1. The PU.1–GATA-1 switch also interacts with another mutually antagonistic pair, 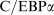
–FOG-1. The latter pair inherits the state of its upstream master genes and further reinforces the decision due to several feedback loops, thereby leading to irreversible commitment. The genetic switch, which handles the erythroid-myeloid lineage decision, is an example of a network that implements both a primed and a committed state by regulating cooperativity through recruitment of cofactors. Perturbing the feedback between the master regulators and downstream targets suggests potential reprogramming strategies. The approach points to a framework for lineage commitment studies in general and could aid the search for lineage-determining genes.

## Author Summary

An important question in developmental biology is how different lineage choices are regulated at the genetic level. Robust lineage decisions are implemented by genetic switches, whereby one set of master genes are ON and another set are OFF, leading to a specific expression pattern of genes for a particular lineage. We develop a computational model to illustrate these principles as applied to the hematopoietic erythroid-myeloid lineage choice, where two master regulator genes, PU.1 and GATA-1, function as a genetic switch. The model, which is based upon known interactions, suggests missing interactions between the master genes, which we hypothesize, so as to reproduce the desired dynamics. Furthermore, there exist feedback interactions between the master genes and their downstream targets. When these are included in the model, the dynamics imply that the feedback is responsible for irreversible commitment. Our results suggest the search for missing interactions between the master genes in terms of a coregulated cofactor. The second important result of the model is that reprogramming irreversible cell fate may be possible by perturbing feedback regulation between the master genes and their downstream targets. Hence, dynamical modeling provides prediction of novel mechanisms and also strategies for reprogramming the fates of cells.

## Introduction

Stem cell fates are decided upon the basis of which genes are turned ON/OFF. However, prior to commitment, it has been observed that many genes are expressed at intermediate or basal levels for the hematopoietic stem cell system [1],[2]. Such “priming” behavior for progenitors could allow for rapid deployment of transcription factors to implement particular genetic programs. In hematopoiesis there exist several lineage branch points with identified key transcription factors and external signals [3]–[5]. A particularly well studied subnetwork is that of PU.1 and GATA-1. It governs the erythroid - myeloid lineages and demonstrates both the commitment as well as priming features [6],[7]. Both PU.1 and GATA-1 are autoregulatory [8],[9], thereby providing stability to their levels, once expressed. PU.1 and GATA-1 also regulate each other in a mutually antagonistic way [10]–[12], such that either of them are expressed exclusively in a fully committed state [7]. Mutual antagonism, an example being the toggle switch [13], enables the selective expression of a gene while suppressing the other. Recent investigations [14],[15] focus on this mechanism to regulate choices of expressed genes as part of the macrophage/neutrophil lineage.

From forced expression studies in both cell lines and primary cells, it is evident that GATA-1 and PU.1 are able to specify erythroid and myeloid cell fates (see [16] and references therein). It is also clear that both GATA-1 and PU.1 cross-antagonize each other's activity. Biochemical studies suggest that in one case this occurs through the inhibition of DNA binding of cognate cis-regulatory motif while in the other case DNA binding is unaffected but the transactivation potential is inhibited [17]. Precisely how GATA-1 and PU.1 then initiate the presumed cascade of transcriptional changes that culminate in the specification of terminally differentiated erythroid and neutrophilic cells is currently unclear and the subject of intense experimental investigation. Global chromatin immunoprecipitation studies will no doubt provide insights into the relevant target genes in both cases. In the case of GATA-1 it is however clear that the situation will be complicated by the occurrence of different GATA-1 complexes which may create both positive and negative transcriptional activity upon GATA-1 itself [18]. Such considerations may be pertinent in the context of GATA-1/FOG-1 interactions, which may either be positive or negative depending on the presence or use of additional partner proteins. How these interactions, which have been documented in fully committed erythroid cells, play out at earlier stages of differentiation at the time of commitment decisions involving PU.1 and GATA-1 is at the moment not clear.

From a dynamical point of view, a biological network, such as the PU.1–GATA-1 genetic switch, which is responsible in determining two different lineages, would be expected to exhibit bistability. In general switch-like behavior can give rise to phenotypic diversity [19],[20], by allowing different states to be sampled. An ultrasensitive [21] switch-like behavior on the other hand lacks built-in memory of the system, and hence is not as robust to fluctuations in the input signal. In [22],[23], bistability, has been shown to occur in mammalian gene networks. Bistability, which is seen to arise from positive feedback in systems [24]–[27], has been explored in several circuits [13],[28],[29], and has also been discussed mathematically [30].

The PU.1–GATA-1 system, which displays switch behavior [31], has encouraged the development of two computational models which describe the effects of autoregulation and mutual antagonism on the dynamics of the transcriptional network [32],[33]. In [32], the authors discuss multistability and priming properties in terms of autoregulatory, cross-inhibitory and cross activation interactions. They show that priming might occur when either both PU.1 and GATA-1 are expressed at very low levels, or, alternatively, at intermediate expression of both genes with stronger cross activation strengths. In [33], using a combination of mathematical modeling and experimental data, it is shown how the PU.1–GATA-1 network encodes the possibility of priming. Assuming that PU.1 and GATA-1 repress each other's expression, the only possible states of the system are either PU.1 at a high level and GATA-1 at a low level, and *vice versa*, or an unstable “progenitor” state at which both PU.1 and GATA-1 are expressed at intermediate levels. The authors showed that autoregulation at both PU.1 and GATA-1 confer stability to the progenitor state.

**Figure 1:**
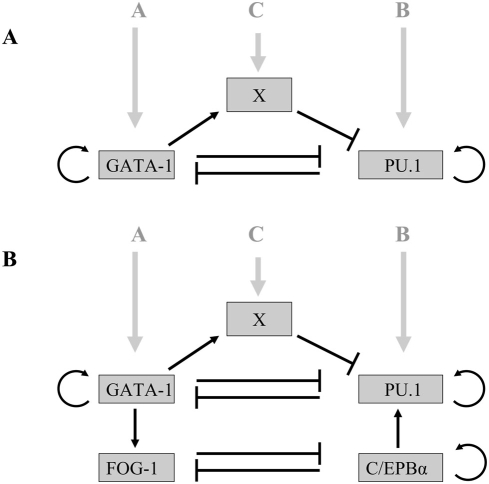
The PU.1–GATA-1 and 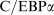
–FOG-1 switches. (A) The PU.1–GATA-1 circuit, showing their auto-regulatory and mutually antagonistic interactions, as well as further interaction through the ‘master regulator gene’ X. The environmental signals into PU.1, GATA-1 and X that integrate the nuclear circuitry with the external environment are denoted A, B and C respectively. (B) The PU.1–GATA-1 switch shown together with the reinforcement from the downstream 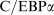
–FOG-1 system with the mutually antagonistic interactions between 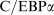 and FOG-1 [34].

Although both models [32],[33] successfully describe the switch-like, priming properties of the network, they assume cooperativity in bindings (Hill coefficients ≥2) between the transcription factors and the genes for bistability to occur. However, recent experiments [12] suggest that mutual regulation of PU.1 and GATA-1 seems to occur through the binding of a PU.1-GATA-1 heterodimer to the PU.1 and GATA-1 genes, with little or no evidence of regulation due to higher order multimers, of PU.1–GATA-1. Furthermore, there is no evidence that the autoregulation at both PU.1 and GATA-1 occurs through the binding of dimers. As we will demonstrate in our model, which is based upon these experimental facts, the nature of the bindings plays an important role—simple heterodimeric repressive bindings between PU.1 and GATA-1 and monomeric autoregulatory bindings do not suffice for bistability (see Text S1 and Figure S1). As a consequence, an additional mechanism must be involved to make this mutually antagonistic pair function as a bistable switch. Several cofactors of PU.1 and GATA-1 are known to bind on their target genes (see *e.g.* [3]–[5] and references therein). We therefore propose the existence of an additional gene X, which is regulated by one of the mutually antagonistic partners, and furthermore is recruited by it, to bind to the other, as a repressor. This mechanism provides the necessary feedback required for bistable behavior. On the other hand, suppression of X (see Figure 1A), leads to a loss of the cooperativity and hence switch-like state, and therefore leads to a primed state.

Hematopoiesis is a hierarchically structured process with a series of progenitors or intermediates which serve as semi-stable and restricted states for future lineage decisions. This organization implies that network information must be handed over from one cell type to another in a way that maintains prior settings and precludes reversibility. Here we have examined the principles of how hand-over and irreversibility might be achieved in the context of the pair of transcription factors 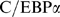 and FOG-1. These factors, which are responsible for eosinophil lineage commitment, are located downstream of GATA-1–PU.1 and are themselves mutually antagonistic [7],[34]. As we will discuss later, the 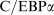
–FOG-1 pair first inherits the state of its master genes, PU.1 and GATA-1, and then further reinforces the decision, by feeding back positively. The positive feedback leads to irreversible commitment. Understanding details of this mechanism therefore provides insights into how the commitment can be reprogrammed.

## Results

### The PU.1–GATA-1 Switch Requires a Connector Gene X

The model for the PU.1–GATA-1 system is based upon assumptions that follow experimental results [7],[12]. Both PU.1 and GATA-1 each undergo positive autoregulation with each protein binding to their respective genes as activators. The mutual antagonism between PU.1 and GATA-1, although achieved by different mechanisms, both involve interactions between the PU.1 and GATA-1 proteins, as well as the ability of the GATA-1–PU.1 heterodimer to bind to each of the genes [12]. At the PU.1 promoter, GATA-1 competes with C-Jun (a co-activator of PU.1), to bind to PU.1 at the PU.1 promoter, leading to the suppression of PU.1. Correspondingly, the GATA-1–PU.1 heterodimer inhibits GATA-1 transcription due to two factors: (i) PU.1 recruits the co-repressor Rb and other chromatin modifying transcription factors and (ii) PU.1 prevents acetylation of GATA-1 by CBP, the latter which is required for erythroid differentiation. For simplicity, we model both these interactions in a similar way; the PU.1-GATA-1 heterodimer binds to both PU.1 and GATA-1 as a repressor.

The equations for PU.1 and GATA-1 protein concentrations, denoted by [P] and [G] respectively, have the form,
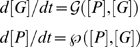
(1)where the functions 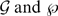 are given in *Methods*. The steady state values of [P] and [G] are obtained by simultaneously solving Equation (1) by setting the right hand sides to zero. In Text S1 (and also Figure S1) we demonstrate that multiple solutions, in particular three states of the system (two stable and one unstable), which are required for bistable behavior, cannot be obtained, based upon these interactions as described in Equation (3) in *Methods*. Therefore, this system, which lacks cooperativity, does not provide a bistable switch. We therefore propose the following mechanism which can provide the necessary cooperativity and hence give rise to bistability. Assume the existence of a gene X, which is induced and subsequently recruited by GATA-1 to bind to PU.1 as a repressive heterodimer. This results in increased cooperativity as it is analogous to a homodimer of GATA-1 binding to PU.1, since X itself is activated by GATA-1. This mechanism which uses X, is in addition to the already existing repressive interaction of the PU.1-GATA-1 heterodimer at the PU.1 regulatory region. In Figure 1A, the network is shown, where A, B and C are the environmental signals into PU.1, GATA-1 and X respectively.

With [X] denoting the concentration of X, we obtain a modified set of equations for the network (see *Methods*):
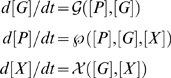
(2)When solving this modified system, one obtains multiple stable states. An analysis of how the curves, d[P]/dt = 0 and d[G]/dt = 0, intersect and give rise to three states, two stable and one unstable, is given in Text S1 (and also Figure S1).

**Figure 2:**
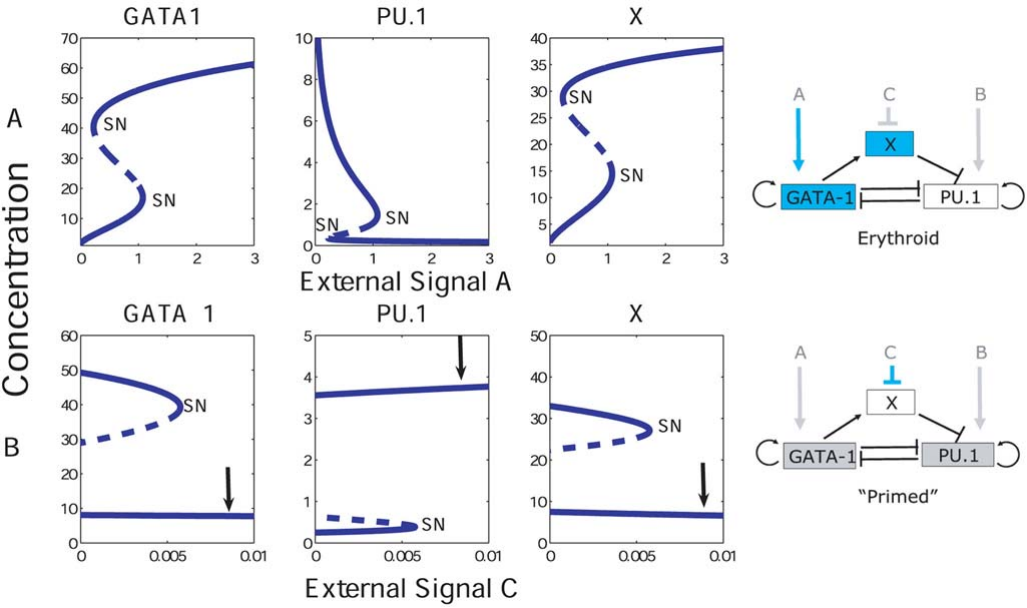
Concentrations of GATA-1, PU.1 and X as functions of the environmental signals A and C. SN denotes saddle nodes and the unstable points are drawn as dotted lines. (A) The system exhibits bistable behavior as a function of A (*B* = 0.5, 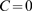). (B) Effects from repressing the X gene through external means (*A* = 0.6, 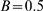). The external factor C reduces the X levels. This in turn reduces the recruitment of the X protein by GATA-1 at PU.1, which relaxes the repression at PU.1. Hence the bistability is lost. Therefore, the system is ‘primed’, through expression of intermediate levels of GATA-1 and PU.1 (as indicated by the arrows). The panels on the right are cartoons of the committed and primed states respectively as a function of the external factors A and C.

In Figure 2 (upper panel) the PU.1, GATA-1 and X steady state concentrations are shown as functions of the environmental signal A, while keeping B at a low level and 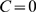. The latter allows X to be fully expressed, since C suppresses the X gene (see *Methods*). As can be seen, the concentrations exhibit bistability/hysteresis behavior with respect to A. It is interesting to consider the primed state, which occurs when both PU.1 and GATA-1 are at intermediate levels. In Figure 2 (lower panel) the PU.1, GATA-1 and X levels are shown for 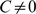, *i.e* when C is allowed to suppress X. All protein levels are here primed at intermediate levels once C crosses a certain threshold. Suppression of X through the external signal C results in the loss of cooperativity by which GATA-1 can bind to PU.1. A similar bistable behavior is obtained for the protein concentrations as functions of B, an external signal that induces PU.1. Simulations performed over a range of parameters indicate that the bistable behavior is a robust dynamical consequence of this basic architecture. One should note that we have chosen a particular scheme, in which GATA-1 causes X to be expressed and further recruits it as a repressor to PU.1. The behavior of the system is symmetric with respect to the directionality of the X gene regulation.

A crucial point is that a generic availability of co-activators is not sufficient to provide the cooperativity that is required for the bistability. The co-activators must be *directly* or *indirectly* induced by GATA-1/PU.1. A major difference with previous models [32],[33], which parameterize cooperativity in terms of Hill coefficients ≥2, is that we make no such assumptions. Our model is based upon the experimentally observed heterodimeric binding for repression and in the absence of any other experimental evidence, we make the simplest assumption, namely, we assume monomeric bindings for activation. However, we make the hypothesis of an additional gene to provide the necessary cooperativity in the network, and hence nonlinearity for bistable behavior. In [32], the authors use heterodimeric binding for repression, but assume dimerization for the autoregulatory interactions, which in their model gives rise to bistability. In comparison, we hypothesize the X gene. However, from a dynamics standpoint, the introduction of the X gene not only renders the system bistable, but very importantly, it also provides a robust mechanism for switching from a primed state to commitment. A mutually antagonistic pair of genes, with high cooperativity such that a bistable state is achieved, can be primed only if the levels of activation are low. The primed state as well as bistability as a function of activation is discussed in the context of macrophage/neutrophil lineage commitment in [14]. At higher levels of activation the system is inherently unstable, and hence priming such a state necessarily requires low levels of input excitation. However, priming levels in hematopoiesis have been observed at 5% to 10% levels of full expression [35], which would argue against inherently large cooperativity. Rather, the default state could be one of low cooperativity, and once the appropriate combinations of external signals come ON, cofactor binding can give rise to cooperativity. This would provide an opportunity for priming at higher levels of expression.

Recent experiments [36] suggest that self-association of GATA-1 is important for erythroid lineage development. However, at this point, it is not clear if dimers of GATA-1 can bind to PU.1 and thereby interrupt both autoregulatory loops. However, we have explored in Text S1 and Figure S9 such a model, which would not require an X gene. Although the self-association of GATA-1 provides the required cooperativity for switch-like behavior, as has just been discussed, it becomes difficult to find a primed state. Hence, there is a trade off between switch-like behavior in an antagonistic system, which in most cases gives widely differing steady states, and a primed state, where the transcriptional factors are at comparatively low levels.

The following scheme for lineage choice for the switch emerges:

- Initially both PU.1 and GATA-1 are expressed at low levels via the external factors A and B, and X is kept at a low level, *i.e.* the system is primed.
- A lineage choice is then made once the inhibition of X is released by the removal of C.

### Reinforcement and Final Commitment with 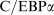 and FOG-1

**Figure 3:**
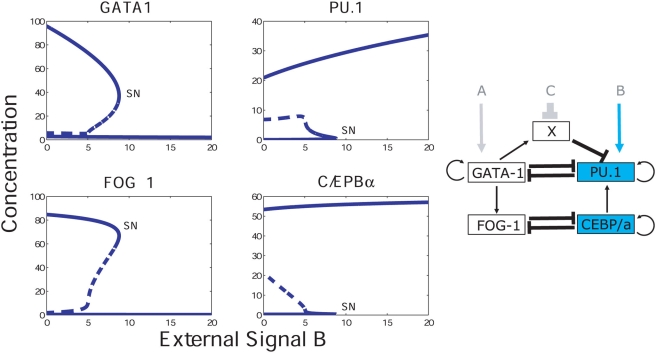
Concentrations of GATA-1, PU.1, FOG-1 and 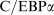 as functions of the environmental signal B (*A* = 1, 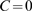) in the case where strong feedback from 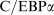 occurs. As can be seen, the feedback induces irreversibility; the switch becomes fully committed and the final concentration levels do not change much, even when the input signal B is removed. Same notations as in Figure 2.

PU.1 and GATA-1 connect to the downstream genes 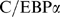 and FOG-1. The latter pair has also been implicated as a mutually antagonistic system, which is responsible for the eosinophil lineage commitment program [7],[34]. Furthermore, FOG-1 is activated by GATA-1 and PU.1 is activated by 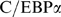 [7]. In addition, 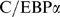 is autoregulatory [37]. The network that emerges from these interactions is displayed in Figure 1B. In addition to the interactions shown, we assume that there are external signals which induce 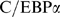 and FOG-1. Due to the lack of any biochemical information about the mechanism of the mutual antagonism between 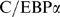 and FOG-1, we assume that they bind as monomers, to each others genes as repressors. We assume that GATA-1 and 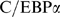 are positive activators, and bind as monomers on the FOG-1 and PU.1 genes respectively. The positive autoregulation of 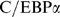 is also assumed to be due to monomeric binding. From these simple assumptions, Equation (4) is modified to Equation (5) augmented with equations for d[F]/dt and d[E]/dt, where [F] and [E] denote the FOG-1 and 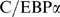 concentrations (see *Methods*). As can be seen in Figure 3, PU.1, GATA-1, 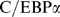 and FOG-1 exhibit a single turning point (unlike Figure 2A with two turning points) as functions of the environmental signal B; [G] drops to low levels at [B] ≈8. Further reduction of [B] has minimal influence of [G], and subsequently remains “locked” at a low value. Thus, the switch remains in the OFF-state ([G] low and [P] high) and retains its commitment—the switch is irreversible. The same holds for the other protein concentrations. This commitment is a consequence of the architecture, and arises due to positive feedback of PU.1 on itself. The latter is a result of two repressions, which “add up” to an activation: PU.1⊣GATA-1, GATA-1→FOG-1, 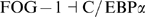, and finally 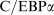
→PU.1. Furthermore since 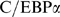 is autoregulatory, it is able to retain high levels even after the environmental factor B is reduced, thereby keeping the PU.1–GATA-1 switch permanently ON.

**Figure 4:**
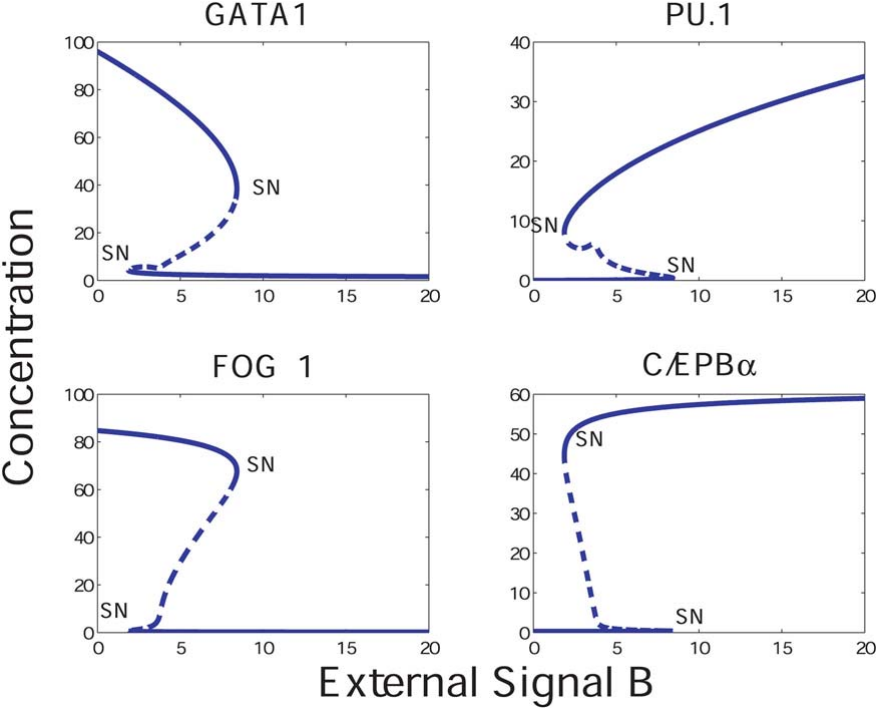
Concentrations of GATA-1, PU.1, FOG-1 and 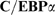 as functions of the environmental signal B for weak feedback from 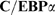 (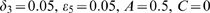). The curves exhibit bistable behavior but not irreversibility. Same notations as in Figure 2.

An important consideration is whether the feedback from 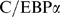 to PU.1 is absolutely essential to provide reinforcement of the commitment decision, and whether the network can be modified to reverse the commitment. Indeed, if the feedback strength from the downstream gene 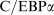 is reduced, then the irreversibility is lost. In Figure 4 we show all the protein levels, the same as in Figure 3, but with a reduced feedback strength from 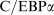
→PU.1. One identifies two turning points, which indicates a reversible switch-like behavior. Similarly, reversible switch-like behavior is obtained if the forward induction of the downstream gene FOG-1 by GATA-1 is weakened. In either case the system can be reprogrammed, from a state of final commitment, which points to possible experiments. The PU.1/GATA-1 switch can be made to be irreversible even without the downstream FOG-1 and 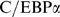 interactions. This can occur if the GATA-1/X complex binds strongly to PU.1 as a repressor (see Text S1 and Figures S2 and S3). However, from a functional perspective, the dynamics of the integrated network, indicates that after the initial decision is communicated downstream to FOG-1 and 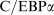, their dynamics signals to their master regulators (PU.1/GATA-1), and this leads to commitment. In this way, there is enough opportunity for the system to abandon commitment at the progenitor stage should the downstream events not take place. Another alternative for lineage commitment is to have each switch in the hierarchy as independently irreversible. This however would require high cooperativity which could be achieved through multimer bindings or by X-like mechanisms. We propose that as a design principle, it is more likely that irreversibility arises only when a secondary decision is made downstream, and is communicated to its upstream master genes, as a signal for final commitment.

We have investigated the effects of various aspects of the architecture on the dynamics of the network; the regulation of the X gene and autoregulation in the 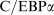
–FOG-1 subnetwork. The X mechanism confers bistability to the PU.1-GATA-1 switch, whereas the 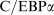
–FOG-1 interaction with PU.1 and GATA-1 accounts for the reiteration of a decision which is taken upstream by the master regulators PU.1-GATA-1. In Text S1 and Figure S4 we describe the role of autoregulation of 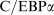 in the irreversible behavior of the switch. We show that reducing autoregulation of 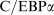 leads to loss of irreversibility. This occurs because, after 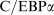 has been induced through suppression of GATA-1/FOG-1, by PU.1, it is unable to maintain itself due to lack of autoregulation on removal of external signal B, and hence cannot provide strong positive feedback to keep PU.1 at a high level. We have also modeled the effect of autoregulation of FOG-1, which (see Text S1 and Figure S5) has the following consequence. Even though high values of PU.1 can switch OFF GATA-1, which inhibits the induction of FOG-1, the autoregulation at FOG-1, can keep it at relatively high levels. Hence, this prevents commitment into the myeloid branch, since 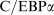 is suppressed. Due to the hierarchical structure of the network, the primed state for PU.1/GATA-1, which is obtained by repressing X, gets inherited by 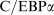
–FOG-1. This occurs since GATA-1 primes FOG-1, which in turn keeps 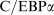 primed. The latter is maintained at a low level, such that it activates PU.1 weakly. Hence X functions as a master regulator, keeping all the components in a primed state.

It is intriguing to consider FOG-1 to be in fact the X gene, since FOG-1 has been found to bind together with GATA-1, at several target genes. We have explored the possibility of FOG-1 playing the role of the X gene (details are discussed in Text S1 and Figures S6, S7, and S8. The network displays switch-like behavior, with respect to signals A and B. This is not very different from the network with the X gene, since the basic architecture remains the same. However, the major difference appears when the issue of priming of the system arises. As we have seen, suppression of X leads to the loss of cooperativity by which GATA-1 can effectively suppress PU.1, and this leads to a primed state. Suppression of FOG-1 however, leads to a completely different response: PU.1, 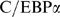 are high and GATA-1, FOG-1 are low. This result is to be expected, since, suppression of FOG-1, relieves suppression of 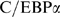, which due to autoregulation, allows it to increase, which in turn activates PU.1. Hence, although FOG-1 provides functionality of the X gene, it is difficult to keep the system primed at low levels for all transcription factors.

## Discussion

We have devised a simple model for the PU.1-GATA-1 genetic switch which, in addition to known interactions, involves a feedforward mechanism through a connector gene X. This mechanism provides the required cooperativity resulting in a bistable switch. In addition, if X is suppressed the cooperativity of the system is lost, and it becomes possible to have both PU.1 and GATA-1 at reasonably expressed levels—the primed state. The network components therefore regulate cooperativity, which can be affected by external signals.

It is interesting to note that, a similar regulatory scheme, in which a connector gene (X) bridges the master regulators, through a feedforward structure [38],[39] and where these master regulators interact directly with each other and are autoregulatory, has in fact been identified in human hepatocytes [40]. Hence, future work could explore computational models, to query such similar architectures.

The second issue is how irreversibility of the erythroid-myeloid lineage switch can be achieved through feedback from other lineage components, namely FOG-1–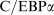. The switch-like behavior exhibited by the PU.1–GATA-1 network is first ‘inherited’ by the downstream mutually antagonistic pair FOG-1–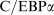 as GATA-1 communicates this decision to FOG-1. Then the positive feedback from 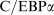 into PU.1 further supports this decision, which leads to an irreversible commitment. In addition, autoregulation of 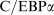 further strengthens this positive feedback. Lineage decisions communicated to downstream genes, which in turn feed back to its master regulators, provides an attractive mechanism for robust commitment from a design principle perspective—unless the downstream program is not fully implemented, the master switch is not irreversibly ON.

The system can be reprogrammed by reducing the feedback from GATA-1 downstream to FOG-1, or by the upstream activation of 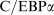 to PU.1. This reduces the strength of the positive feedback of PU.1 on itself and hence the genetic switch can be made reversible. As discussed in [6], specific combinations of transcription factors give rise to distinct lineages in the hematopoietic system. This is achieved in the present model due to the interaction of the inherent positive and negative feedbacks which give rise to stable dynamical states. Hence, GATA-1 and FOG-1 give rise to erythroid/megakaryocytic lineage, PU.1 and C/EBP give rise to the myeloid lineage. Our model can also allow for intermediate levels of GATA-1 and high levels of 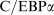, which specifies eosinophils. This can be achieved through reduced feedback from 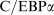 into PU.1, allowing 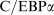 to reach high levels of autoregulation, and exciting GATA-1 through the external signal A. Also, in support of our model is the observation of the reprogramming of B-cells into myeloid lineages by over-expressing 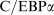 [41],[42]. Referring to Figure 3, when 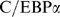 is ON, PU.1 is induced.

Identification of the X gene should be possible through loss-of-function studies of the PU.1–GATA-1 system. Combining ChIP-chip with gene expression experiments [43] for PU.1 and GATA-1 would be crucial. Specifically, with the particular scheme that we model, in the erythroid lineage, X is fully expressed, whereas in the myeloid lineage, X is shut down. It would seem obvious that a strong candidate for the X gene could be none other than FOG-1, since GATA-1 and FOG-1, together regulate several downstream targets [7]. However, as discussed in the previous section, a model with FOG-1 as the X gene, even though allowing the system to be bistable, may make the primed state more difficult to achieve.

Mutual antagonism among pairs of genes has been suggested as a general mechanism for lineage commitment [5],[7]. In addition there are several examples [6],[14] where upstream pairs of antagonistic master genes prime and regulate downstream genes which are also antagonistic. For instance, EKLF–Fli-1 [4],[5], inherits the PU.1/GATA-1 decision, and further regulates the erythroid lineage. Using our current model as an illustration, we hypothesize that lineage commitment in an architecture which consists of layers of antagonistic pairs of genes connected such that the lower levels reinforce the upper level decisions, results in positive feedback, giving robust lineage commitment.

One issue not addressed here are the effects of noise. Stochasticity in gene expression has now been both theoretically as well as experimentally explored and been shown to be due to both intrinsic as well as extrinsic factors [44]–[46]. Recent investigations have also explored stochasticity in genetic switches [47],[48], which show the effects of switching dynamics due to molecule number fluctuations. One of our future goals is to investigate the effects of noise on the irreversibility of the PU.1-GATA-1 switch.

## Methods

The network dynamics is modeled using the Shea-Ackers formalism [49]–[52], which is based upon a thermodynamic model for transcription. We assume that the process of transcription and translation can be lumped together. Hence, our models consist of transcription factors, and their interactions in a genetic control network. The steady state solutions are analyzed as a function of the network parameters, in particular, the environmental factors. We assume that the concentrations are in dimensionless units and the kinetic constants are in units of 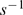, and the Michaelis-Menten constants are dimensionless.

### The PU.1–GATA-1 System with Heteromeric Bindings

The dynamical equations corresponding to Equation (1) are given by
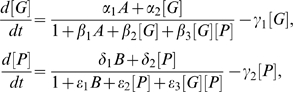
(3)where we denote by A and B the environmental factors acting on GATA-1 and PU.1. A and B integrate the switch with its environment. Parameter values are given in Table 1.

**Table 1:** Parameter values used in the dynamical equations.

**A**	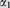	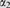	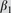	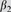	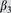	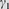	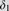	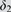	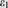	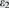	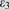	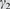	
	1	0.25	1.0	0.25	1	0.01	1	0.25	1.0	0.25	1	0.01	
**B**	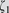	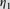	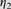	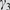	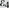								
	0.01	0.01	10	0.01	0.13								
**C**	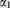	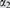	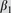	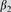	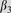	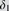	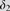	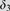	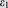	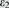	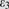	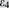	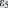
	1	0.25	1.0	0.25	1	1	0.25	0.27	1.0	0.25	1	2	0.27
	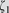	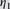	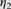	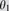	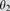	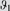	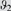	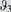	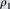	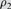	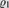	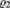	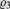
	0.01	0.01	10	1	0.075	1.0	0.075	1	1	0.025	1.0	0.025	1

(A) Values used for the PU.1–GATA-1 network in Equation 3. (B) Values of the additional parameters used for the PU.1–GATA-1 network including the gene X in Equation 4. For Figure 2A, 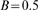, and for Figure 2B, 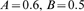. (C) Parameter values used for the 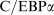
–FOG-1 network in Equation (5). 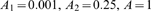, the degradation parameters 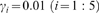. For Figs. 3 and 4 the parameter 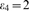 is used.

### The PU.1–GATA-1 System with Connector Gene X

The dynamical equations corresponding to Equation (3) are given by
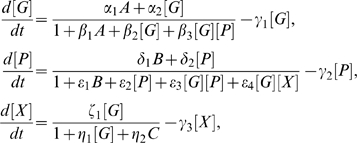
(4)


Here we have assumed that an external signal C regulates X independently of PU.1 and GATA-1, and in particular can be used to repress it. Hence when C is not present, X is fully expressed, *i.e.* when GATA-1 itself is at a high level. Alternatively, C could also be chosen as an activator of X, which means that it is required for the expression of X, and hence also required to be present for repression of PU.1, by regulated recruitment by GATA-1.

### Including the FOG-1–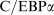 Loop

The dynamical equations corresponding to the network in Figure 1B are given by
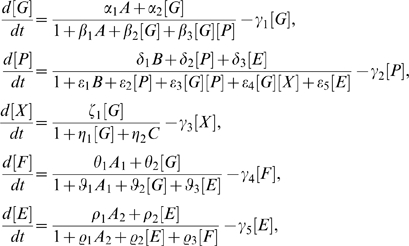
(5)


In Equation (5), the external signals to [F] and [E] are A_1_ and A_2_ respectively and the parameters values are displayed in Table 1. We did not introduce a corresponding gene X for the FOG-1 and 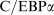 interaction, since the PU.1–GATA-1 switch behavior is inherited by the FOG-1–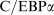 mutually antagonistic network.

Simulations of the differential equations were implemented using MATLAB software (The Mathworks) and the Systems Biology Workbench (SBW/BioSPICE) tools [53]: JDesigner, and Jarnac. The bifurcation diagrams were generated using Oscill8 [54].

## Supporting Information

Figure S1 Effects of the gene X. The nullclines, d[P]/dt = 0 and d[G]/dt = 0, from Eqs. (3,4), with parameters in Table 1 (A, B), and with the external signals A = 0.75, B = 0.5. A single stable point (s) of intersection between d[P]/dt and d[G]/dt is obtained if X is not present (Eq. (1)). When X is included (Eq. (2)), the co-operativity shifts the d[P]/dt = 0 nullcline downwards to intersect with d[G]/dt = 0 at three points, 2 stable s and one unstable u, therefore exhibiting bistable behavior (C = 0).
(0.03 MB EPS)

Figure S2 Concentrations of GATA-1, PU.1 and X as functions of the environmental signal A, when the binding strength of the repressive heterodimer GATA-1-X is made to bind strongly to the PU.1 regulatory region (ε_4_ = 0.25). The curves exhibit irreversibility.
(0.02 MB EPS)

Figure S3 Concentrations of GATA-1, PU.1 and X as functions of the environmental signal B, when the binding strength of the repressive heterodimer GATA-1–X is made to bind strongly to the PU.1 regulatory region (ε_4_ = 0.25). The bistable curves are not irreversible.
(0.03 MB EPS)

Figure S4Concentrations of GATA-1, PU.1, FOG-1 and 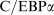 as functions of the environmental signal B, without autoregulation of 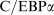. The curves exhibit bistable behavior but not irreversibility, since 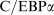 cannot remain high on removal of B, thereby unable to provide positive feedback to PU.1.(0.04 MB EPS)

Figure S5Concentrations of GATA-1, PU.1, FOG-1 and 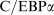 as functions of the environmental signal B, including autoregulation of 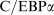. The curves exhibit bistable behavior but not irreversibility because even when PU.1 suppresses GATA-1, and hence FOG-1 by increasing B, FOG-1 continues to be high because of autoregulation, and this leads to continued repression of 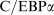. The latter, therefore, cannot provide positive feedback to PU.1, and hence the irreversibility is lost.(0.04 MB EPS)

Figure S6Concentrations of GATA-1, PU.1, FOG-1 and 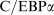 as functions of the environmental signal A, with FOG-1 playing the role of the X gene. The curves exhibit bistable irreversible behavior.(0.07 MB EPS)

Figure S7Concentrations of GATA-1, PU.1, FOG-1 and 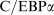 as functions of the environmental signal B, with FOG-1 playing the role of the X gene. The curves exhibit bistable irreversible behavior.(0.06 MB EPS)

Figure S8Concentrations of GATA-1, PU.1, FOG-1 and 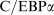 as functions of the environmental signal C, with FOG-1 playing the roles of the X gene. C is used to repress FOG-1, which is expected to “prime” the system. However, suppression of FOG-1 leads to increased levels of 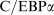 and subsequently PU.1, which indicates that, unlike in the “X” system, here, the network is unable to be primed, where all concentrations are at an intermediate level.(0.04 MB EPS)

Figure S9 Concentrations of GATA-1, PU.1 as functions of the environmental signal A for the case when GATA-1 dimers associate with PU.1 to repress each other's expression, as well as auto-regulate GATA-1. For low values of A, the system is unable to be primed, and in fact as shown by the arrows, the bistable switch ultimately becomes irreversible, if GATA-1 dimers self associate even stronger.
(0.03 MB EPS)

Text S1 (0.07 MB PDF)
