# Supplementary material for: Computational Modeling of the Hematopoietic Erythroid-Myeloid Switch Reveals Insights into Cooperativity, Priming, and Irreversibility
Source: PLoS Comput Biol. 2009 Jan 23;5(1):e1000268. doi: 10.1371/journal.pcbi.1000268 (PMC2613533; doi:10.1371/journal.pcbi.1000268)
Supplement: Text S1 — (0.07 MB PDF) [file pcbi.1000268.s010.pdf]

Supporting Information

Computational modeling of the hematopoietic erythroid-myeloid switch reveals insights into co-operativity, priming and irreversibility

Vijay Chickarmane, Tariq Enver and Carsten Peterson

In this Supporting Information we provide the following additional details:

- Bistability of the PU.1–GATA-1 system – mathematical aspects.
- An irreversible bistable PU.1–GATA-1 switch.
- Is it necessary for C/EBPα to be autoregulating for irreversibility of the network?
- Consequences of autoregulation of FOG-1 for the PU.1–GATA-1 switch.
- Can FOG-1 play the role of the X gene?
- Circuit properties of the GATA-1–PU.1 switch due to self-association of GATA-1.

Bistability of the PU.1–GATA-1 system

Mathematical properties. We explore the consequences of the particular type of activation and repressive bindings that we have assumed for PU.1 and GATA-1 on the dynamics of the network. We show that the PU.1–GATA-1 system as described with heterodimeric bindings for repression and monomeric bindings for activation, cannot display bistable behavior. This can be seen from solving Eq. (3)[1] for the steady state concentrations [P] and [G]. One gets

$$\begin{aligned} [G] &= \frac{\alpha_1 + \alpha_2[G]}{1+\beta_1+\beta_2[G]+\beta_3[G][P]}, \\ [P] &= \frac{\delta_1+\delta_2[P]}{1+\epsilon_1+\epsilon_2[P]+\epsilon_3[G][P]}, \end{aligned} \tag{S1}$$

where the α_i and δ_i $(i = 1, 2)$ are renormalized constants by dividing with the degradation constants γ_i, and for simplicity $A, B = 1$. Solving for [P] in the first equation and then replacing for [P] in the second, leads to the following fifth order polynomial equation for [G],

$$\begin{aligned} p([G]) &= \epsilon_3{\beta_2}^2[G]^5 + (\epsilon_2{\beta_2}^2 - 2\beta_2\epsilon_3 s_1 + s_2\beta_2\beta_3 - \delta_1{\beta_3}^2)[G]^4 \\ &+ (-2s_1\beta_2\epsilon_2 + {s_1}^2\epsilon_3 - 2\alpha_1\beta_2\epsilon_3 - s_1 s_2\beta_3)[G]^3 \\ &+ (\epsilon_2{s_1}^2 - 2\alpha_1\beta_2\epsilon_2 + 2s_1\alpha_1\epsilon_3 - s_2\beta_3\alpha_1)[G]^2 \\ &+ (2s_1\alpha_1\epsilon_2 + {\alpha_1}^2\epsilon_3)[G] + \epsilon_2{\alpha_1}^2 \end{aligned} \tag{S2}$$

where $s_1 = \alpha_2 - 1 - \beta_1$ and $s_2 = \delta_2 - 1 - \epsilon_1$. The five solutions to $p([G]) = 0$ are difficult to obtain analytically. For the bistability behavior, we require exactly *3 positive solutions*. The other two solutions could be either negative or a complex conjugate pair. To analyze the qualitative nature of the solutions we use the *Descartes Sign Rule* [2], which states: Counting the number of sign changes (N) that occur as we read each of the terms of a polynomial, including the ones with missing exponent, one can at most have N, $N-2$, $N-4$ ... positive solutions (the number of solutions are reduced by 2, since one must account for complex conjugate pairs with positive real parts). By inspection of Eq. (S2), the coefficients multiplying $[G]^5$ and $[G]^0$ are positive. This implies that sign changes can occur for the coefficients of $[G]^4$, $[G]^3$, $[G]^2$ and $[G]^1$. There are therefore 16 possibilities for these sign changes. For each of these we apply Descartes Rule. For example, in the case where the coefficients for $[G]^4$ and $[G]^2$ are both negative, we obtain the following structure for the polynomial equation, where only the signs of the coefficients are displayed

$$p([G]) = [G]^5 - |(...)|[G]^4 + |(...)|[G]^3 - |(...)|[G]^2 + |(...)|[G] + |(...)|. \tag{S3}$$

There are in total 4 sign changes. Therefore a maximum of $4, 2$ or 0 positive roots are possible. If we reverse the sign, and use $-[G]$ in Eq. (S3), then the maximum number of **negative** roots is obtained. In this case we have a maximum of one negative root. Hence, if

[1] Equation, table and figure numbers refer to the main text, whereas enumerations with prefix "S" can be found in this Supplementary Text.

[2] Anderson, B., Jackson, J. & Sitharam, M. 'Descartes' rule of signs revisited. Amer. Math. Monthly **105**, 447-451 (1998).

we had 3 positive roots, one negative root, that would leave us with one root to account for, which would not come into either class. This therefore excludes the possibility of obtaining exactly 3 positive roots. An examination of the remainder of the cases, also shows that there is not a single exception, where the number of positive roots is exactly 3. The system therefore cannot exhibit bistability.

Nullcline analysis. In Fig. S1 we show the steady state values of PU.1 vs GATA-1 as described by Eqs. (3) and (4). In Fig. S1, upper panel, we show how the nullclines, d[P]/dt=0 (blue line) and d[G]/dt=0 (black line) as described by Eq. (1) intersect at a single point. In this case only a single stable solution is possible. This is the *primed* state, which represents small levels of both PU.1 and GATA-1 to be expressed. Taking into account the X gene interaction, the nullcline d[P]/dt=0 is modified, as described in Eq. (4). As shown in Fig. S1, lower two panels, it intersects the nullcline d[G]/dt=0 at three points. Two of these can be verified to be stable (s) and one as unstable (u) – the system therefore is bistable. The increased cooperativity through interaction with the X gene, results in pulling down the d[P]/dt=0 nullcline (blue line to the red dotted line). These graphs were obtained for fixed values of A, B and C.

An irreversible bistable PU.1–GATA-1 switch

In Fig. S2 we show the steady state values of PU.1, GATA-1 and X, as a function of the environmental factor A for parameter values described in Table 1 (A, B), with external signal $B = 0.5$ and $\epsilon_4 = 0.25$. As can be seen the system is irreversible, *i.e*, when A crosses a certain threshold, GATA-1, X switches ON, and remain locked at high levels thereafter. This occurs when the binding strength of the GATA-1-X repressive heterodimer to the PU.1 regulatory region is increased. This is because, even on removal of the A signal, low amounts of this heterodimer are sufficient to suppress PU.1 due to the increased binding strength, and in addition GATA-1 is autoregulatory, which ensures that GATA-1 retain high levels, and hence PU.1 can never be turned ON. However, in Fig. S3, we display similar curves with respect to environmental signal B for parameter values described in Table 1 (A, B) and with external signal A=0.6 and ϵ_4=0.25, which shows that irreversibility is not obtained. Increasing the repression on PU.1 through increased binding of the repressive heterodimer makes it harder for the switch to be irreversible with respect to B. This is why we have mainly focused on the dynamical features of the network with respect to environmental signal B throughout the paper, since it is much easier to obtain irreversibility with respect to A.

Is it necessary for C/EBPα to be autoregulating for irreversibility of the network?

Autoregulation of C/EBPα has a very important role to play for the irreversibility of the switch. In Fig. S4 (for parameter values see Table 1 (C), and for signal $A = 0.75$, ρ_2=0, ϱ_2=0, Eq. 5), the irreversibility is lost due to the loss of autoregulation of C/EBPα. The irreversibility occurred due to the ability of C/EBPα to autoregulate itself and provide the positive feedback to PU.1, thereby keeping it a high level, even when the signal B is removed.

However, on loss of the autoregulation, C/EBPα, falls to a low level, once B decreases (since GATA-1 and FOG-1 increase, and this leads to suppression of C/EBPα), and the feedback to PU.1 is insufficient to keep it at a high level.

Consequences of autoregulation of FOG-1 for the PU.1/GATA-1 switch

We now consider autoregulation of FOG-1 as a possibility and ask what effect does this have on the network dynamics. To describe this effect we have to modify the equation for $d[F]/dt$, to be,

$$\frac{d[F]}{dt} = \frac{\theta_1 A_1 + \theta_2[G] + \theta_3[F]}{1 + \vartheta_1 A_1 + \vartheta_2[G] + \vartheta_3[E] + \vartheta_4[F]} - \gamma_4[F], \tag{S4}$$

In Fig. S5 (all parameters are as in Table 1(C), and the external signal $A = 1$, $\theta_3 = 0.025, \vartheta_4 = 0.025$), we see that the irreversibility of the switch is once again lost due to the ability of FOG-1 to keep itself at a high level, and thereby keep C/EBPα at a low level. Since the latter provides positive feedback to PU.1, the result is that the system becomes reversible. Hence, the network architecture suggests that for the PU.1–GATA-1 network to be irreversible, C/EBPα must be autoregulating, whereas as FOG-1 should not be autoregulating.

Can FOG-1 play the role of the X gene?

Since FOG-1 has been found to bind together with GATA-1, at several target genes, we explore the possibility of FOG-1 playing the role of the X gene. To describe this mathematically, Eq. 5 in the main text can be replaced by,

$$\begin{aligned}
\frac{d[G]}{dt} &= \frac{\alpha_1 A + \alpha_2[G]}{1 + \beta_1 A + \beta_2[G] + \beta_3[G][P]} - \gamma_1[G], \\
\frac{d[P]}{dt} &= \frac{\delta_1 B + \delta_2[P] + \delta_3[E]}{1 + \epsilon_1 B + \epsilon_2[P] + \epsilon_3[G][P] + \epsilon_4[G][F] + \epsilon_5[E]} - \gamma_2[P], \\
\frac{d[F]}{dt} &= \frac{\theta_1 A_1 + \theta_2[G]}{1 + \vartheta_1 A_1 + \vartheta_2[G] + \vartheta_3[E] + \vartheta_4 C} - \gamma_4[F], \\
\frac{d[E]}{dt} &= \frac{\rho_1 A_2 + \rho_2[E]}{1 + \varrho_1 A_2 + \varrho_2[E] + \varrho_3[F]} - \gamma_5[E],
\end{aligned} \tag{S5}$$

where the parameter values are given in table S1.

In Figs. S6 and S7, the curves show the irreversible switch-like behavior, of the network with respect to signals A and B. This behavior is not very different from the network with the X gene, since the basic architecture remains the same. However, the major difference appears when the issue of priming the system arises. As we have seen, suppression of X leads to the loss of cooperativity by which GATA-1 can effectively suppress PU.1, and this leads to a primed state. Suppression of FOG-1 however, leads to a completely different response. In

Fig. S8, the concentrations are shown as functions of the signal C, which suppresses FOG-1. As we see, at some threshold of C, only one possible state is obtained: PU.1, C/EBPα are high and GATA-1, FOG-1 are low. This result is to be expected, since, suppression of FOG-1, allows C/EBPα to rise, which in turn activates PU.1. Hence although FOG-1 provides functionality of the X gene, priming the system becomes an issue.

Circuit properties of the GATA-1–PU.1 switch due to self-association of GATA-1

In this section we model the PU.1-GATA-1 switch assuming self-association of GATA-1 [1]. We therefore assume (i) a GATA-1 dimer positively regulates GATA-1 (autoregulation), (ii) a GATA-1 dimer binds to PU.1 to suppress both genes–PU.1 and GATA-1. Eq.(3) in the main text can be replaced by,

$$\begin{aligned}\frac{d[G]}{dt} &= \frac{\alpha_1 A + \alpha_2 [G]^2}{1 + \beta_1 A + \beta_2 [G]^2 + \beta_3 [[G]^2 [P]} - \gamma_1 [G], \\ \frac{d[P]}{dt} &= \frac{\delta_1 B + \delta_2 [P]}{1 + \epsilon_1 B + \epsilon_2 [P] + \epsilon_3 [G]^2 [P]} - \gamma_2 [P],\end{aligned} \tag{S6}$$

where the parameter values are given in table S1.

In Fig. S9, the steady state values of GATA-1 and PU.1 show switch-like behavior with respect to the external signal A (with $B = 0$), which clearly shows that self-association of GATA-1 is sufficient to provide the required cooperativity for bistability. However, for low values of the external signal A, the switch cannot be "primed", as seen in the low values of GATA-1 and relatively large values of PU.1. In fact if the binding strengths of the GATA-1 dimer to either the GATA-1 increase (strong self-activation) or PU.1 operator is increased (strong repression)(corresponding to α_2, ϵ_3 respectively), the switch becomes increasingly irreversible. The self-association provides strong nonlinearity, however making it increasingly difficult to find a primed state, in which neither GATA-1, nor PU.1 are at high levels.

[1] Shimizu R, Trainor CD, Nishikawa K, Kobayashi M, Ohneda K (2007) Gata-1 self-association controls erythroid development in vivo. J Biol Chem 282: 15862-15871.

TABLES

**A**												
α_1	α_2	β_1	β_2	β_3	δ_1	δ_2	δ_3	ϵ_1	ϵ_2	ϵ_3	ϵ_4	ϵ_5
1	0.25	1.0	0.25	1	1	0.25	1	1.0	0.25	1	0.75	1.0
θ_1	θ_2	ϑ_1	ϑ_2	ϑ_3	ϑ_4	ρ_1	ρ_2	ϱ_1	ϱ_2	ϱ_3		
1	0.1	1.0	0.1	1	10	1	0.1	1.0	0.1	1		
**B**												
α_1	α_2	β_1	β_2	β_3	δ_1	δ_2	ϵ_1	ϵ_2	ϵ_3			
1	0.045	1	0.045	0.1	1	0.1	1	0.1	0.0075			

Table S1: **A** Parameters used for Fig. S6 (B=1), Fig. S7 (A=1) and Fig. S8 (A=1, B=1). A_1 =0.01, A_2 =0.015 and the degradation parameters γ_i=0.01 (i=1:5). **B** Parameters used for Fig. S9 (B=0), γ_i=0.01 (i=1,2).
